# Supplementary material for: Neural Networks Modeling for Prediction of Required Resources for Personalized Endourologic Treatment of Urolithiasis
Source: J Pers Med. 2022 May 12;12(5):784. doi: 10.3390/jpm12050784 (PMC9143218; doi:10.3390/jpm12050784)
Supplement: Supplementary file 1 [file jpm-12-00784-s001.zip › jpm-1664710-supplementary.pdf]

Supplementary Materials

Table S1. An overview about pre-processing, model training, split of data into training, validation and test sample, training stop criteria, selection of thresholds for the reject option and final test of the model.

	Description		*Remarks*
Input normalization and encoding	Continuously distributed variables were standardized having mean 0 and unit variance	Discrete variables were encoded using one hot encoding	Pre-processing of input data before model training
Split of data into training, validation and test samples	Full sample (n=401) was randomly split into a training sample (n=360) and test sample (n=41). 10-fold cross-validation was used for model training using 10% of training data as validation set each. No data of the test sample was used for model training.	After model training, two thresholds for the reject options were estimated to maximize negative and positive predictive values and to minimize number of unpredicted cases.	These thresholds were applied and neg. and pos. predictive values were computed in the training samples and finally evaluated in the independent and randomly selected test sample (n=41, Tab.3)
Model training	ADAM optimization algorithm		ADAM is a stochastic gradient descent using an adaptive learning rate that is invariant to diagonal rescaling of the gradients.
Training stopping criteria	Based on the network loss with a relative change of loss	Maximal training rounds <= 500 rounds	To mitigate overfitting, early stopping approach and cross-validation were used. Additionally, L2-regularization techniques were also used.
Model performances	Negative and positive predictive values were computed based on 10-fold cross-validation in all models (Tab.2).		
Final test	Trained neural network models with two thresholds allowing the reject option were independently tested in the test sample. Negative and positive predictive power and percentage of unclassified subjects were compared in the training and test sample. The performances were similar suggesting no overfitting (Tab.3)		

Table S2: Overview of model performances with various combinations of cut-offs for the neural network model ('use of laser lithotripsy').

Lower cut-off c1	Upper cut-off c2	Negative predictive value (%)	Positive predictive value (%)	Percentage of unpredicted cases (PUC) (%)	Remarks
0.5	0.5	92%	91%	0%	This corresponds to the model without using a reject option.
0.4	0.6	93%	91%	3%	NPV and PPV did not change much, PUC slightly increased.
0.3	0.7	93%	92%	7%	NPV remained stable, PPV was slightly increasing. PUC slightly increased.
0.2	0.8	95%	94%	18%	NPV and PPV were increasing. PUC increased.
0.1	0.9	97%	indeterminate	40%	NPV was increasing, PPV was could not be computed. PUC significantly increased.
0.12	0.78	97%	94%	34%	Final decision about the cut-offs and - correspondingly percentage of unpredicted cases - was done after intensive discussions with the medical experts about their suggestions concerning NPV and PPV.

NPV and PPV were 92% and 91% for the endpoint 'use of laser lithotripsy' before application of the reject option (corresponds to the initial model, Tab. 2, c1=c2=0.5). NPV and PPV were increasing to 97% and 94% after application of the reject option (corresponds to the model with reject option, Tab. 3). The cost for this improvement is that 34% of all patients did not receive a prediction. Both selected cut-offs c1 and c2 were applied in a test sample to check whether results remain stable (Tab. 3).

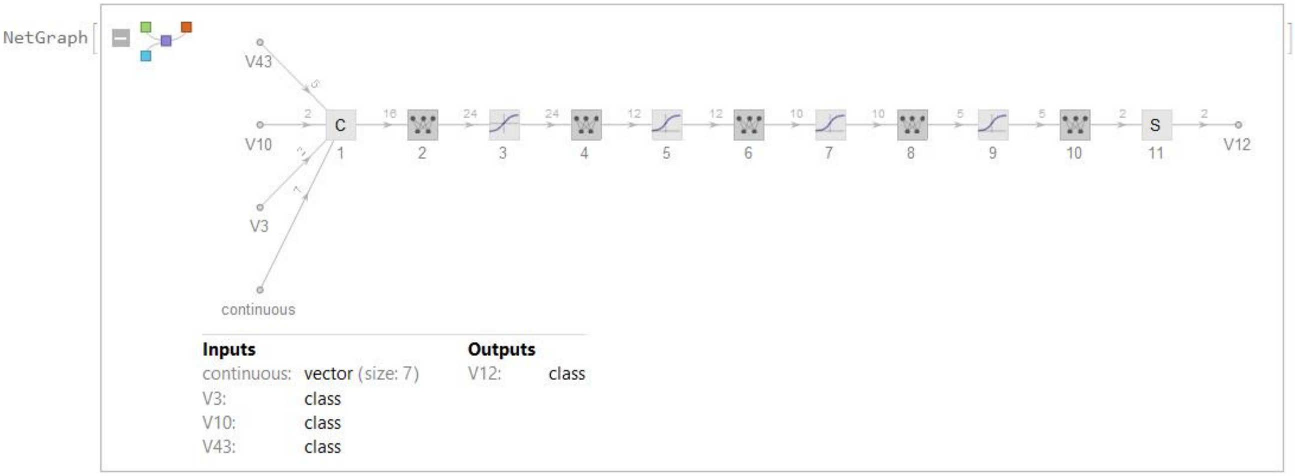

Figure S1: Network architecture of the neural network including all layers and activation functions for use of laser lithotripsy. Layers and activation functions are illustrated and enumerated in the figure (1-11). The following section describes type and structure of each layer and activation function by using these numbers: 1) Catenation layer: Input vector consists of 3 discrete and 7 continuously distributed variables which were catenated by a catenate layer 2) Linear layer, i.e. a real matrix with dimensions 24x20 3) Hyperbolic tangent was used activation function 4) Linear layer with dimensions 12x24 5) Logistic sigmoid as activation function 6) Linear layer with dimension 10x12 7) Logistic sigmoid as activation function 8) Linear layer with dimensions 5x10 9) Logistic sigmoid 10) Linear layer with dimensions 2x5), 11) Softmax layer.
